# *Apprends-moi l'art des petits pas:* Levodopa, Carbidopa Intestinal Gel plus Entacapone

**DOI:** 10.1007/s00702-023-02625-6

**Published:** 2023-04-28

**Authors:** Wolfgang H. Jost

**Affiliations:** Parkinson-Klinik Ortenau, Kreuzbergstr. 12-16, 77709 Wolfach, Germany

**Keywords:** Advanced Parkinson’s disease, Intrajejunal pump therapy, Levodopa, Levodopa pump, LECIG

## Abstract

We are always looking for the big breakthrough, ideally a cure for our advanced Parkinson’s disease (aPD) patients. As long as this does not happen we must optimize the existing therapy, because many small steps may also lead to success. This also applies to the levodopa pump: Certainly, a very good therapy, but with small problems that we have to optimize. This involves, for example, the weight and volume of the previous pump. One possibility is to use the proven triple combination as intestinal gel, thereby increasing the levodopa plasma concentration. Increasing the levodopa plasma concentration enables the reduction of the given levodopa dose and hence the size of the pump. To learn more about the triple combination as intestinal gel the ELEGANCE study was started. This study is a prospective non-interventional study of the long-term effectiveness and safety of levodopa–entacapone–carbidopa intestinal gel (LECIG) in patients with aPD in routine care. This observational study is designed to collect data on the use of the drug Lecigon® in daily clinical practice. The study is intended to supplement the results of previous clinical studies with clinical data in routine medical care, collected from approximately 300 patients.

## Introduction

We need to pay special attention to our advanced Parkinson’s disease (aPD) patients, that stage of the disease which we still cannot treat adequately with oral medication. Today we are in the fortunate position of having a plethora of advanced therapy options available that allow our patients to be treated well for many years if all these options are also used. We have the worldwide accepted deep brain stimulation along with systems for applying levodopa or a dopamine agonist subcutaneously or intrajejunally via a pump system. The pump therapies are reversible options and can be removed at any time whereas deep brain stimulation will remain forever in the brain of the patients.

## LECIG—Levodopa, Entacapone, Carbidopa Intestinal Gel

We are always looking for the big breakthrough, ideally a cure. As long as this does not succeed, however, we must optimize the existing therapy, because many small steps may also lead to success. This also applies to the levodopa pump: Certainly, a very good therapy, but with small problems that we have to optimize. This involves, for example, the weight and volume of the previous pump.

**Fig. 1:**
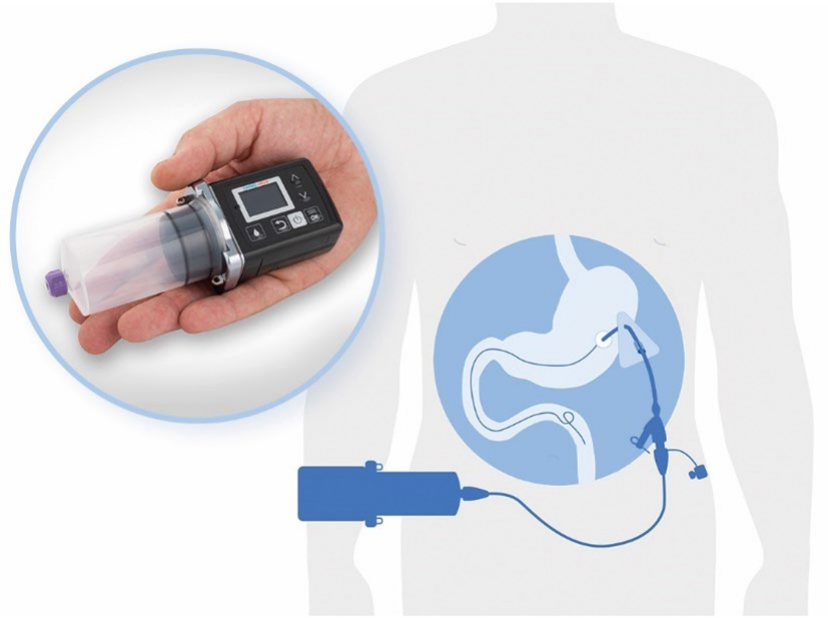
The CRONO LECIG pump system and the position of the tube system

One problem can be solved by reducing the weight of the pump. But just reducing the size/weight of the pump would lead to the need for constantly changing the cartridge. In this case, less is more: a small, lightweight pump can be realized by a reduced dose. Coming from the combination levodopa/carbidopa, what would be the best option to reduce the dose? As levodopa is still the most important and most effective drug therapy for PD the best way forward is to stay with it and reduce the total dose, by reducing the problems of a short half-life and the resulting short duration of action. Combining levodopa and carbidopa is a first, indispensable step to increase the half-life. But levodopa is not only metabolized directly to dopamine. It is also metabolized by a second pathway. Addition of the cathecol-*O*-methyltransferase (COMT) inhibitor entacapone, blocking levodopa’s second-largest pathway, leads to less levodopa conversion to 3-*O*-methyldopa (3-OMD), thereby increasing the levodopa plasma concentration. Increasing the levodopa plasma concentration enables the reduction of the given levodopa dose and hence the size of the pump used to apply the triple combination. The proven triple combination of levodopa, entacapone and carbidopa (LEC) was previously only available orally. Since February 2021 it is also available as an intestinal gel formulation (IG) via a pump system. The 3 active ingredients (LEC) are continuously delivered via the pump system directly into the duodenum or jejunum, the site of absorption. The modern pump system (CRONO LECIG) is smaller, lighter and quieter (Fig. 1). This offers users more convenience and flexibility in everyday life, as well as discreet wearing of the device.

## Current knowledge about levodopa pump therapies and ongoing studies

For many years levodopa/carbidopa intestinal gel (LCIG) has shown that levodopa pumps may have benefits for patients with advanced PD (aPD) that last for 12 months or more (Antonini et al. 2021). LCIG has been available for many years and is known to help patients by reducing ‘off’-time. Despite this, less is known about how long the benefits of LCIG last. Summarizing all information available on the long-term use of LCIG shows that when patients have been taking LCIG for at least 12 months, they have 2–4 h less ‘off’-time each day than they did before starting the LCIG treatment. This effect is maintained for 3–5 years after starting LCIG treatment. There were no unexpected side effects with long-term use of LCIG. The time not spent in ‘off’ may allow people with aPD to increase their independence in daily activities (Antonini et al. 2021).

Infusion of LCIG is an effective treatment for patients with aPD suffering from motor fluctuations despite optimal oral levodopa therapy (Pauls et al. 2021). Previous studies have demonstrated beneficial effects, with lessening of motor fluctuations and increases in ON time without troublesome dyskinesia and reduced ‘off’-time. Non-motor symptoms also improved, resulting in an overall improvement in quality of life (Pauls et al. 2021).

To compare the pharmacokinetics of levodopa when administered as LECIG or as LCIG, a randomized, open-label, 2-day crossover study was conducted to measure systemic levodopa exposure over 14 h with a 20% reduced LECIG infusion versus the patient's usual LCIG infusion dose (Senek et al. 2017). Eleven patients (aged ≥ 30 years) with idiopathic aPD participated in the study: They had received stable LCIG treatment for at least 30 days and had not been treated with entacapone within 3 months before the study. Patients were randomized to one of two treatment sequences and received either LCIG followed by LECIG or vice versa for two consecutive days.

Both treatments were administered via the same gastrojejunostomy tube using an infusion pump for 14 h. LECIG doses were 80% (*n* = 5) or 90% (*n* = 6) of the usual LCIG morning dose, 80% of the LCIG continuous (maintenance) dose, and 80% of the extra doses. The primary endpoint was systemic levodopa exposure. Other endpoints included patient motor function as assessed by Treatment Response Scale (TRS) scores, pharmacokinetics of levodopa, carbidopa, 3-*O*-methlydopa, and entacapone, and safety through adverse event monitoring.

Although a lower dose of levodopa was administered in LECIG, systemic levodopa exposure did not differ significantly between treatments, suggesting that the bioavailability of levodopa over a 14-h infusion in LECIG was comparable to that in LCIG (Senek et al. 2017). TRS scores showed no difference between treatment groups despite the lower levodopa dose administered as LECIG (Senek et al. 2017). From this study, it was concluded that the increased bioavailability of levodopa in the presence of entacapone means that lower total levodopa doses can be administered with LECIG to achieve therapeutically effective plasma concentrations.

In addition, 3-OMD plasma concentrations were increased by 22% when switching from LECIG to LCIG but decreased by 35% when switching from LCIG to LECIG. Therefore, treatment with LECIG may reduce exposure to potentially harmful levodopa metabolites.

Initial clinical experience with LECIG infusion in Sweden was evaluated in an observational study of 24 PD patients (12 of whom were switched from LCIG infusion to LECIG infusion) at Uppsala University Hospital, Sweden. The study also described patient-reported outcomes and their perceptions of the therapy (Öthman et al. 2021). All patients treated with LECIG infusion were assessed for eligibility and then followed up according to the usual clinical routine at Uppsala University Hospital: one visit, either outpatient or inpatient, every six months, according to the Swedish National Guidelines for the Treatment of PD. Patients completed questionnaires to determine whether their symptoms had changed, how user-friendly the pump was, what activities of daily living the patients performed, what their health-related quality of life was, and, for patients who were switched from LCIG to LECIG, to make a comparison between these two therapies. The 24 patients (11 women and 13 men) included in the analysis had a mean age of 71.5 years at the start of LECIG infusion, a mean duration since PD diagnosis of 15.5 years, and a mean LECIG treatment duration of 305 days (Öthman et al. 2021). Patients switched from LCIG to LECIG used a median of 100% of the previous morning LCIG dose at baseline, whereas the continuous infusion rate was reduced to 76% of the previous LCIG dose at LECIG infusion.

Most patients reported an improvement in their ability to perform activities of daily living and in their self-rated quality of life with LECIG infusion therapy, and a high proportion of patients (70%) who had not previously used levodopa infusion (*n* = 10) felt an improvement in their symptoms (Öthman et al. 2021). Among patients who had switched from LCIG to LECIG, the effect of LECIG infusion on PD symptoms was most often perceived to remain the same (45%) compared with LCIG—which was to be expected, given LECIG’s ability to deliver therapeutically effective plasma levodopa concentrations despite a lower total levodopa dose. Patients also reported that they were generally satisfied with the ease of use and smaller size of the CRONO LECIG pump compared to the standard LCIG pump (Öthman et al. 2021).

## ELEGANCE

The ELEGANCE study is a prospective non-interventional study of the long-term effectiveness and safety of levodopa–entacapone–carbidopa intestinal gel (LECIG) in patients with aPD in routine care (Lecigon® 2023). This observational study is designed to collect data on the use of the drug Lecigon® in daily clinical practice and is intended to supplement the results of previous clinical studies with clinical data in routine medical care, collected from approximately 300 patients. The ELEGANCE study (NCT05043103) has been established to collect real-world data on the efficacy and safety of LECIG in clinical practice use at centers around Europe. ELEGANCE is an international, prospective, non-interventional, observational study. The planned total number of patients to be recruited to the study is ~ 300 across all countries and will be followed for 24 months or until study discontinuation.

Study centers will offer participation to all adult patients with advanced PD who have severe motor fluctuations and hyperkinesia or dyskinesia despite taking optimized or transdermal PD therapy and who have been prescribed treatment with LECIG as part of routine clinical practice. Subjects can be de novo patients or those who switch from another infusion therapy.

The primary endpoints are to evaluate the long-term efficacy and safety of LECIG over 24 months. The efficacy evaluations will include effect on motor symptoms (change from baseline to 24 months in daily OFF time (MDS-UPDRS IV) and Motor aspects of experiences of daily living (MDS-UPDRS II scores), required levodopa dose, the use of other PD medications, Clinical and Patient Global Impression and the satisfaction with LECIG treatment.

Adverse events (AEs) will be monitored for evaluation of safety. AEs of special interest include dopaminergic side effects, peripheral neuropathy and Vitamin B12 deficiency.

Secondary endpoints are effect on non-motor symptoms (change from baseline in Non-Motor Symptom Scale Score, Parkinson's disease sleep scale-2 and MDS-UPDRS lb scores), change from baseline in Parkinson's Disease Questionnaire total score (PDQ-8 or PDQ-39) and healthcare resource utilization (complications of PD leading to hospitalization; problems with medication or device).

Subject to receipt of marketing authorization for LECIG, it is planned that ELEGANCE will be undertaken in ~ 16 countries and that around 15 centers will participate throughout Germany and Austria, two of the first countries to have launched LECIG, with the aim of recruiting ~ 75 patients. It is furthermore planned that the following countries will also participate in ELEGANCE once marketing authorization is received: Belgium, Bulgaria, Croatia, France, Hungary, Ireland, Italy, Netherlands, Romania, Slovakia, Slovenia, Spain, UK.

The data generated from ELEGANCE will provide valuable real-world information on the efficacy and safety of LECIG. It will also provide information on patient perspectives of this treatment and its impact on healthcare utilization that can help inform clinical decisions and policy making (Lecigon® 2023).

## Peripheral neuropathy

Peripheral neuropathy is a common neurological problem defined as a dysfunction of sensory, motor, and autonomic nerves (Paul et al. 2020). The presence of peripheral neuropathy has recently been noticed in PD. This comorbidity is of concern as it increases the burden on patients whose motor functions are previously compromised. Some studies have established links between prolonged L-dopa exposure and prevalence with increased levels of homocysteine and methylmalonic acid as pathological underlying mechanisms (Paul et al. 2020). Vitamin B12 and cobalamin deficiencies have also been implicated as drivers of peripheral neuropathy. Accumulation of phosphorylated α-synuclein is another central feature in peripheral neuropathy and urgently justifies exploration via large cohort studies. Importantly, these underlying mechanisms have been linked to peripheral denervation. Potential treatments for peripheral neuropathy targeting B12 deficiencies and the use of COMT inhibitors (Paul et al. 2020).

In the ELEGANCE study peripheral neuropathy was chosen as AE of special interest as it is known that the development of peripheral neuropathy is a potential complication during LCIG therapy (Lecigon® 2023). The mechanisms are not fully understood, but levodopa is involved in the metabolization of the B-vitamins, and high doses of levodopa with low levels of B-vitamins and/or high levels of homocysteine are associated with a higher risk for polyneuropathy in PD patients. Inhibitors of catechol-*O*-methyltransferase (COMT) have been used as an oral therapy adjunctive to levodopa since the early 2000s. COMT inhibitors decrease the peripheral metabolization of levodopa and allow higher amounts of levodopa to reach the brain for conversion to dopamine (Lecigon® 2023). Therefore, combining oral levodopa with a COMT inhibitor may potentiate levodopa’s effect by treating wearing-off fluctuations, but this strategy may also cause dyskinesias. Interestingly, COMT inhibitors also prevent the methionine cycle from using B-vitamins to form homocysteine (Lecigon® 2023).

## Data Availability

The empirical data referred to in this paper are available on request from the corresponding author, but are not public due to privacy restrictions.
